# Left Main Coronary Artery Spasm During Cryoballoon Ablation for Atrial Fibrillation: A Case Report and Literature Review

**DOI:** 10.7759/cureus.51902

**Published:** 2024-01-08

**Authors:** Amith Reddy Seri, Fahimeh Talaei, Mahmoud Ibrahim, Mustafa Hassan

**Affiliations:** 1 Internal Medicine, McLaren Flint, Michigan State University (MSU) College of Human Medicine, Flint, USA; 2 Cardiology, McLaren Flint, Michigan State University (MSU) College of Human Medicine, Flint, USA

**Keywords:** case report, st segment elevation, coronary artery spasm, cryoballoon ablation, atrial fibrillation

## Abstract

Although phrenic nerve and esophageal injury are commonly known risks associated with cryoablation, there is limited literature regarding coronary artery spasm (CAS), a serious and potentially fatal complication of cryoablation. We report the case of a 68-year-old Caucasian female who developed a left main CAS with a significant hemodynamic compromise during cryoablation. The patient, with a history of hyperlipidemia, hypertension, and symptomatic persistent atrial fibrillation, was admitted for elective catheter ablation for atrial fibrillation. During the ablation of the left superior pulmonary vein (LSPV), the patient developed severe hypotension and bradycardia. The patient's monitor revealed ST elevation, confirmed by a 12-lead ECG. Immediate coronary angiography revealed the left main coronary spasm, which improved with nitroglycerine administration with resolution of ST elevation and return of the patient’s hemodynamics to stability.

The patient’s left main CAS was induced by cryoablation of LSPV. Literature on atrial fibrillation ablation-induced CAS is scant, but a Japanese study has shown that it occurs more commonly in cryoablation than in radiofrequency, hot balloon, or laser ablation. The study showed LSPV as the most common site of ablation right before the spasms happened. Further studies about this topic are needed to delineate further the risk factors and the precautions that could prevent CAS. In the meantime, prompt recognition and appropriate intervention are critical for a good patient outcome.

## Introduction

Pulmonary vein isolation (PVI) is a common procedure to treat atrial fibrillation. Well-known adverse effects of cryoablation are phrenic nerve injury and esophageal ulceration [1,2]. There have been very few reports of coronary artery spasm (CAS), which could potentially be a life-threatening complication, as an adverse outcome of cryoablation [3]. The few available reports have been mostly in Japanese patients, who are known to have a higher prevalence of vasospastic angina (VSA) when compared to other ethnic groups [4-6]. To our knowledge, there has been only one case report of CAS in a Caucasian male patient following radiofrequency catheter ablation and no case report of periprocedural CAS in a Caucasian female patient undergoing cryoablation [7]. Here, we report the case of a 68-year-old Caucasian female who, while undergoing catheter cryoablation for persistent atrial fibrillation, developed a severe left main coronary spasm with significant hemodynamic compromise. This article was previously posted to Authorea as a preprint in April 2023.

## Case presentation

**Figure 1:**
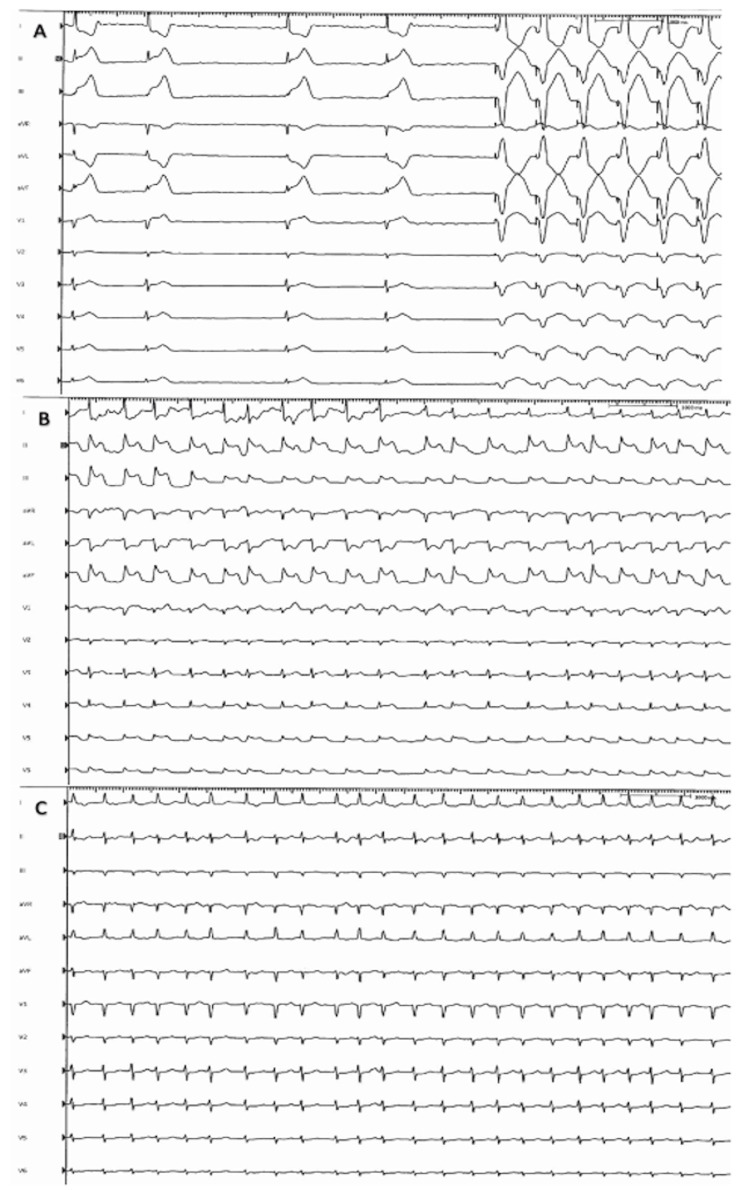
ECG of the patient A: 12-lead ECG showing bradycardia initially followed by ventricular paced rhythm;  B: ECG showing global ST-segment elevation; C: ECG showing resolution of ST-segment elevation after nitroglycerin administration

**Figure 2:**
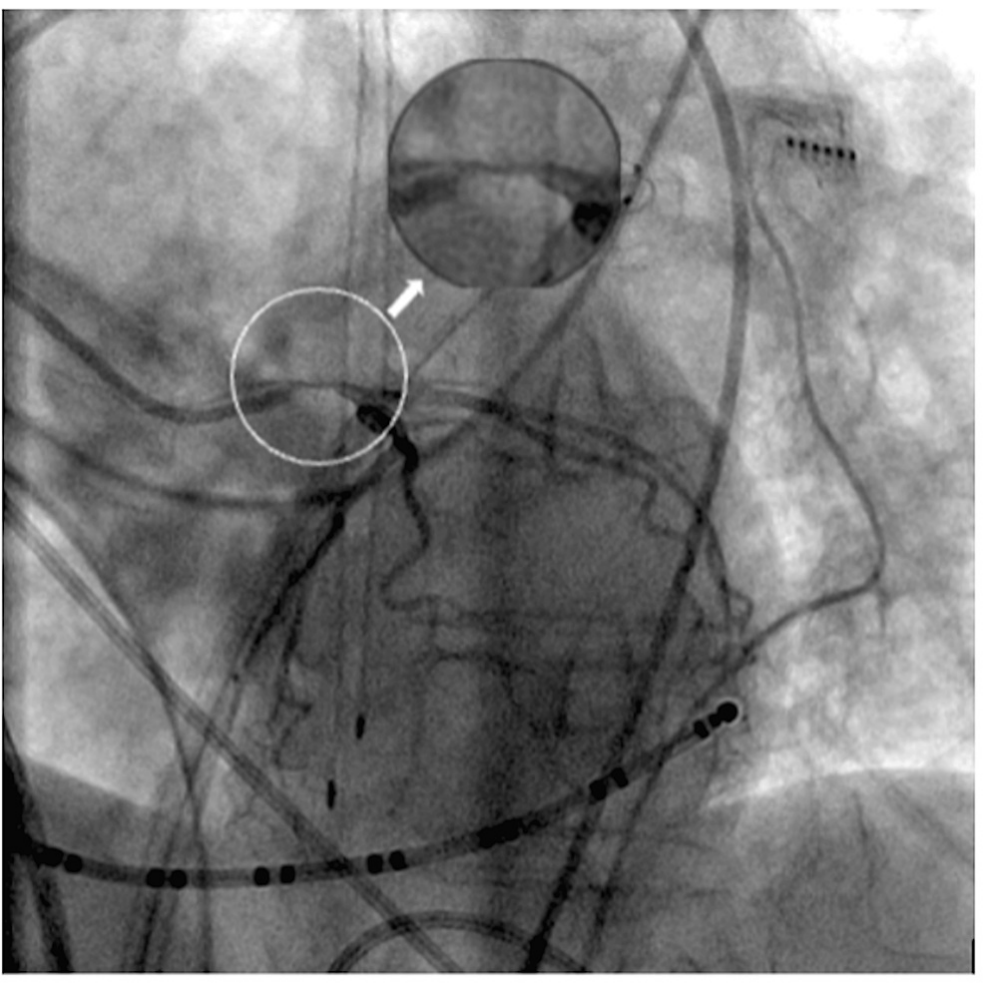
Left heart coronary angiogram showing significant spasm of the left main coronary artery

**Figure 3:**
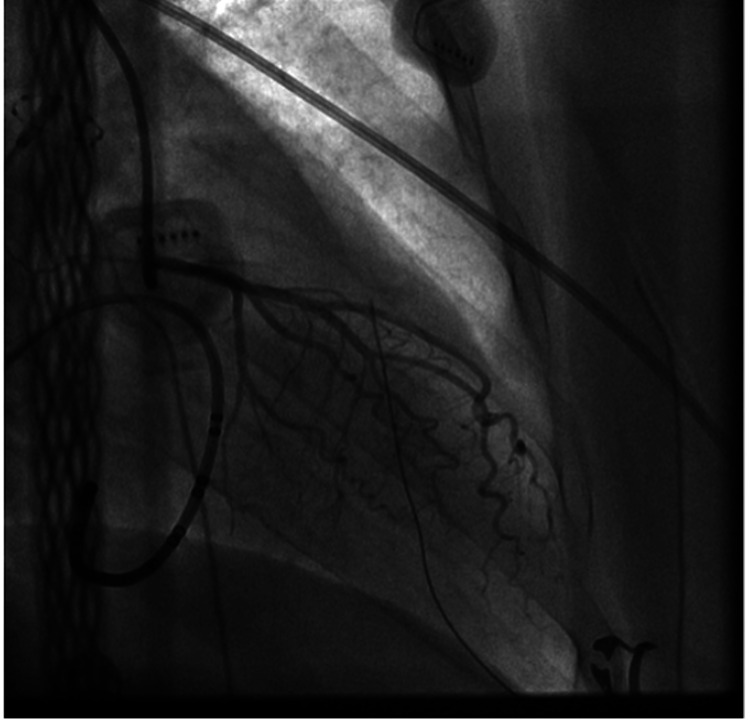
Left heart coronary angiogram showing resolution of the spasm after intracoronary nitroglycerin

A 68-year-old Caucasian woman with a history of hyperlipidemia, hypertension, and symptomatic persistent atrial fibrillation who had failed cardioversion was brought in for catheter ablation. She has no history of coronary artery disease. A transeptal puncture was performed, and the patient was properly anticoagulated. The left superior pulmonary vein (LSPV) was the first vein to be ablated. The cryoballoon was inflated for three minutes, and the lowest temperature achieved was 48^o^C, following which the patient developed severe hypotension (systolic BP in the 50s mmHg) and profound bradycardia with the heart rate down to the 20s. The patient required temporary pacing after failing to respond to atropine (Figure 1 A). Cardiac tamponade was excluded. The 12-lead ECG revealed global ST-segment elevation (Figure 1 B). Immediate coronary angiography revealed a significant spasm of the left main coronary artery with no signs of air embolism (Figure 2). The right coronary artery showed no evidence of spasm. After nitroglycerine administration, the vasospasm completely resolved (Figure 3), improving the patient’s clinical condition. Blood pressure returned to baseline, and the EKG showed resolution of the ST elevation and bradycardia (Figure 1 C). The procedure was resumed with no further complications.

## Discussion

In this report, we describe the case of a Caucasian female patient who developed a left main CAS during cryoablation of the LSPV for the treatment of persistent atrial fibrillation. Coronary artery spasm during cryoablation-PVI was described in several case reports, mostly in the Asian population [8,9]. To the best of our knowledge, this is the first reported case in a Caucasian female. Additionally, most of the coronary spasms were reported in the right coronary artery, and very few cases described left main coronary spasms with such severe hemodynamic effects.

Lehrmann et al. were the first to report a case of CAS in a male. In their report, the EKG showed a pattern of left main stem occlusion while performing right superior pulmonary vein cryoablation [3]. A multicenter, large-scale study in the Japanese population by Nakamura et al. on CAS related to atrial fibrillation ablation reported a higher prevalence of CAS in cryoablation-PVI (0.34%) compared to radiofrequency, hot balloon, or laser balloon PVI ablation (0.04%, 0%, and 0%, respectively). The same study reported LSPV as the most common site of ablation (64% in cryoablation and 75% in radiofrequency ablation) right before spasms happened [5]. Interestingly, in this study, 100% of patients with ST-elevation during LSPV cryoablation showed EKG changes in inferior leads, and 98% of all CAS cases related to all atrial fibrillation ablation approaches were male.

There are two possible mechanisms for a CAS during atrial fibrillation ablation. The first possible mechanism is that the application of a cryoablation catheter may have caused a direct cooling injury to the adjacent coronary arteries [8,10]. This is supported by the fact that CAS has been reported to occur near the cooling site after the application of a cryoablation catheter [9,11]. There is also available data revealing the occurrence of CAS during hypothermia therapy with ice packs and chilled intravenous infusion after successful cardiopulmonary resuscitation, which supports the first mechanism [12]. It is possible that blood cooled by the cryoballoon in the left atrium flows through the coronary arteries and stimulates the coronary endothelium, causing CAS.

Alternatively, the second possible mechanism, which was proposed in radiofrequency ablation, is a possible autonomic nerve activity imbalance leading to CAS. Epicardial sites near the right inferior pulmonary vein are associated with a ganglionated plexus (GP), the so-called right lower GP. Endocardial radiofrequency ablation can affect the epicardial GP through a thermal injury that may cause an imbalance in autonomic nervous activity, frequently stimulating the parasympathetic nerve, which could, in turn, induce vasospasm of the coronary artery [13]. The latter theory was used to describe the CAS happening far from the site of ablation in the Asian population [14].

Since ST elevation happened when we delivered cooling energy adjacent to the LSPV and responded immediately to intracoronary nitroglycerine, we believe that the mechanism causing the severe CAS was most probably cryoenergy-induced blood cooling, causing transient constriction in the left main coronary artery. Lehrmann et al. suggested that there might be a racial difference in the pathophysiology of CAS happening during cryoablation between the Asian and Caucasian ethnicities. Although the case by Lehrmann et al. presented with a near-fatal CAS induced by cryoballoon ablation [3], the patient in the present case was diagnosed in the early phase, which enabled us to proceed with cryoablation after CAS.

## Conclusions

Coronary artery spasms can occur during cryoballoon ablation in patients with no prior history of vasospasms or no prior smoking history. A 12-lead EKG should be continuously monitored throughout the procedure for any ischemic changes. If any ischemic changes are noted to be persistent, consider coronary angiography. Further studies about this topic are needed to further identify the risk factors and precautions that could prevent CAS.
